# Supplementary material for: Hymenolepis diminuta Reduce Lactic Acid Bacterial Load and Induce Dysbiosis in the Early Infection of the Probiotic Colonization of Swiss Albino Rat
Source: Microorganisms. 2022 Nov 24;10(12):2328. doi: 10.3390/microorganisms10122328 (PMC9785584; doi:10.3390/microorganisms10122328)
Supplement: Supplementary file 1 [file microorganisms-10-02328-s001.zip › Supplementary material.pdf]

Table S1. Database showing matching for the strain S10

Sl. No.	Organism Name	Accession No.	% Match
1	Lactobacillus brevis ATCC 14869 = DSM 20054 16S ribosomalRNA	NR_116238.1	99.37%
2	Lactobacillus brevis strain ATCC 14869 16S ribosomal RNA	NR_044704.2	98.90%
3	Lactobacillus senmaizukei DSM 21775 = NBRC 103853 16Sribosomal RNA	NR_114251.1	97.67%
4	Lactobacillus hammesii strain type strain: TMW 1.1236 16Sribosomal RNA	NR_042243.1	97.67%
5	Lactobacillus yonginensis strain THK-V8 16S ribosomal RNA	NR_109452.1	97.55%
6	Lactobacillus cerevisiae strain TUM BP 140423000-2250 16Sribosomal RNA	NR_158030.1	97.46%
7	Lactobacillus senmaizukei DSM 21775 = NBRC 103853 strainL13 16S ribosomal RNA	NR_041584.1	97.42%
8	Lactobacillus koreensis JCM 16448 strain DCY50 16S ribosomalRNA	NR_116854.1	97.38%
9	Lactobacillus parabrevis strain LMG 11984 16S ribosomal RNA	NR_042456.1	97.26%
10	Lactobacillus spicheri strain LTH 5753 16S ribosomal RNA	NR_025579.1	97.13%

Table S2. Database showing matching for the strain S13

Description	Max Score	Total Score	Query Cover	E value	Per. Ident	Acc. Len	Accession
Lactobacillus johnsonii strain CIP 103620 16S ribosomal RNA, partial sequence	987	987	57 %	0.0	88.46%	1543	NR_117574.1
Lactobacillus taiwanensis strain BCRC 17755 16S ribosomal RNA, partial sequence	987	987	57 %	0.0	88.46%	1526	NR_044507.1
Lactobacillus gasseri ATCC 33323 = JCM 1131 16S ribosomal RNA, complete sequence	981	981	57 %	0.0	88.34%	1573	NR_075051.2
Lactobacillus gasseri strain CIP 102991 16S ribosomal RNA, partial sequence	981	981	57 %	0.0	88.34%	1529	NR_117573.1
Lactobacillus hominis DSM 23910 = CRBIP 24.179 16S ribosomal RNA, partial sequence	981	981	57 %	0.0	88.34%	1565	NR_125548.1
Lactobacillus gasseri ATCC 33323 = JCM 1131 16S ribosomal RNA, complete sequence	981	981	57 %	0.0	88.34%	1572	NR_041920.1
Lactobacillus johnsonii strain ATCC 33200 16S ribosomal RNA, partial sequence	976	976	57 %	0.0	88.22%	1487	NR_025273.1
Lactobacillus colini strain 111144-L1 16S ribosomal RNA, partial sequence	953	953	61 %	0.0	86.63%	1566	NR_156075.1
Lactobacillus rodentium strain MYMRS/TLU1 16S ribosomal RNA, partial sequence	887	887	57 %	0.0	86.34%	1436	NR_133768.1
Lactobacillus jensenii strain ATCC 25258 16S ribosomal RNA, partial sequence	865	865	57 %	0.0	85.87%	1496	NR_025087.1

Table S3. Database showing matching for the strain S14

Sl. No.	Organism Name	Accession No.	% Match
1	Lactobacillus johnsonii strain 1696 16S ribosomal RNA gene.	MT597568.1	99.85%
2	Lactobacillus johnsonii strain 1000 16S ribosomal RNA gene.	MT585638.1	99.85%
3	Lactobacillus johnsonii strain 681 16S ribosomal RNA gene.	MT585442.1	99.85%
4	Lactobacillus johnsonii strain 680 16S ribosomal RNA gene.	MT585441.1	99.85%
5	Lactobacillus johnsonii strain 667 16S ribosomal RNA gene.	MT585433.1	99.85%
6	Lactobacillus johnsonii strain 666 16S ribosomal RNA gene.	MT585432.1	99.85%
7	Lactobacillus johnsonii strain 658 16S ribosomal RNA gene.	MT585425.1	99.85%
8	Lactobacillus johnsonii strain 1447 16S ribosomal RNA gene.	MT573842.1	99.85%
9	Lactobacillus johnsonii strain 1029 16S ribosomal RNA gene.	MT573563.1	99.85%
10	Lactobacillus johnsonii strain 7991 16S ribosomal RNA gene.	MT538892.1	99.85%

Table S4. Database showing matching for the strain S17

Description	Max Score	Total Score	Query Cover	E value	Per. Ident	Acc. Len	Accession
Lactobacillus reuteri DSM 20016 16S ribosomal RNA, partial sequence	2636	2636	99 %	0.0	99.72%	1569	NR_075036.1
Lactobacillus reuteri DSM 20016 16S ribosomal RNA, partial sequence	2623	2623	99 %	0.0	99.51%	1571	NR_119069.1
Lactobacillus reuteri strain NBRC 15892 16S ribosomal RNA, partial sequence	2604	2604	98 %	0.0	99.72%	1501	NR_113820.1
Lactobacillus caviae strain MOZM2 16S ribosomal RNA, partial sequence	2518	2518	99 %	0.0	98.39%	1487	NR_157747.1
Lactobacillus frumenti strain TMW 1.666 16S ribosomal RNA, partial sequence	2423	2423	99 %	0.0	97.08%	1561	NR_025371.1
Lactobacillus pontis strain LTH 2587 16S ribosomal RNA, partial sequence	2418	2418	99 %	0.0	97.02%	1568	NR_036788.2
Lactobacillus reuteri strain F275 16S ribosomal RNA, partial sequence	2405	2405	99 %	0.0	96.96%	1535	NR_025911.1
Lactobacillus vaginalis strain ATCC 49540 16S ribosomal RNA, partial sequence	2399	2399	99 %	0.0	96.99%	1541	NR_041796.1
Lactobacillus oris strain DSM 4864 16S ribosomal RNA, partial sequence	2346	2346	99 %	0.0	96.17%	1512	NR_026309.1
Lactobacillus panis strain DSM 6035 16S ribosomal RNA, partial sequence	2327	2327	99 %	0.0	96.14%	1490	NR_026310.1

Table S5. Database showing matching for the strain S27

Sl. No.	Organism Name	Accession No.	% Match
1	Lactiplantibacillus plantarum strain CIP 103151 16Sribosomal RNA	NR_104573.1	96.81%
2	Lactiplantibacillus plantarum strain JCM 1149 16Sribosomal RNA	NR_117813.1	96.81%
3	Lactiplantibacillus plantarum strain NRRL B-1476816S ribosomal RNA	NR_042394.1	96.81%
4	Lactobacillus pentosus strain 124-2 16Sribosomal RNA	NR_029133.1	96.81%
5	Lactiplantibacillus plantarum strain NBRC 15891 16S ribosomal RNA	NR_113338.1	96.74%
6	Lactiplantibacillus plantarum strain NBRC 15891 16S ribosomal RNA	NR_112690.1	96.74%
7	Lactobacillus paraplantarum strain DSM 10667 16S ribosomal RNA	NR_025447.1	96.74%
8	Lactiplantibacillus plantarum strain JCM 1149 16S ribosomal RNA	NR_115605.1	96.74%
9	Lactiplantibacillus plantarum subsp. argentoratensisstrain DKO 22 16S ribosomal RNA	NR_042254.1	96.16%
10	Lactobacillus fabifermentans strain DSM 21115 16S ribosomal RNA	NR_113339.1	96.16%

Table S6. Database showing matching for the strain S29

Description	Max Score	Total Score	Query Cover	E value	Per. Ident	Acc. Len	Accession
Lactobacillus johnsonii strain CIP 103620 16S ribosomal RNA, partial sequence	2782	2782	99%	0.0	99.87%	1543	NR_117574.1
Lactobacillus taiwanensis strain BCRC 17755 16S ribosomal RNA, partial sequence	2776	2776	99%	0.0	99.80%	1526	NR_044507.1
Lactobacillus gasseri ATCC 33323 = JCM 1131 16S ribosomal RNA, partial sequence	2754	2754	99%	0.0	99.54%	1572	NR_041920.1
Lactobacillus gasseri ATCC 33323 = JCM 1131 16S ribosomal RNA, complete sequence	2754	2754	99%	0.0	99.54%	1573	NR_075051.2
Lactobacillus gasseri strain CIP 102991 16S ribosomal RNA, partial sequence	2723	2723	98%	0.0	99.53%	1529	NR_117573.1
Lactobacillus johnsonii strain ATCC 33200 16S ribosomal RNA, partial sequence	2719	2719	97%	0.0	99.66%	1487	NR_025273.1
Lactobacillus hominis DSM = 23910 CRBIP 24.179 16S ribosomal RNA, partial sequence	2686	2686	99%	0.0	98.75%	1565	NR_125548.1
Lactobacillus colini strain 111144-L1 16S ribosomal RNA, partial sequence	2527	2527	99%	0.0	96.83%	1566	NR_156075.1
Lactobacillus rodentium strain MYMRS/TLU1 16S ribosomal RNA, partial sequence	2423	2423	94%	0.0	97.08%	1436	NR_133768.1
Lactobacillus iners strain DSM 13335 16S ribosomal RNA gene, partial sequence	2364	2364	99%	0.0	94.91%	1539	NR_036982.1

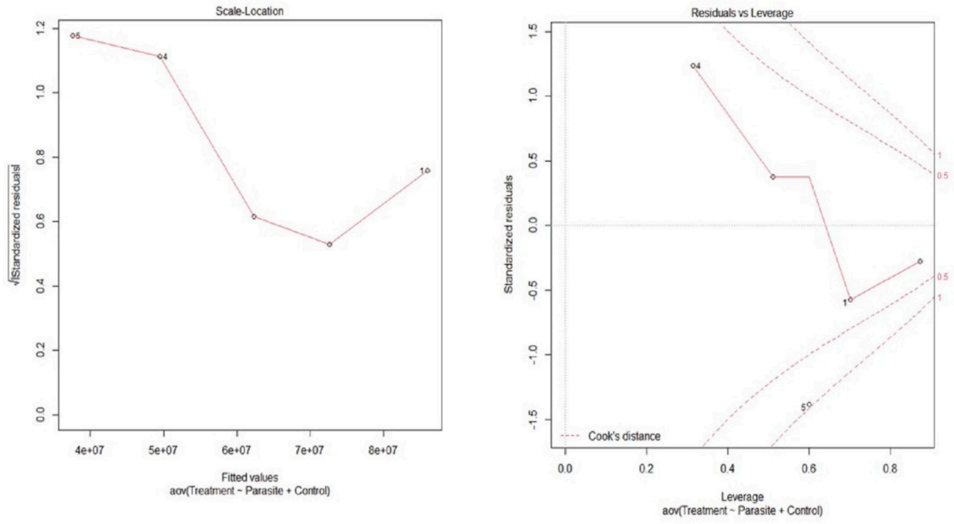

(a) (b)

Figure S1. Detection of outlier in the LAB load: (a) shows the fifth datapoint, i.e., the mean LAB load with respect to parasitic load of 18, is a potential outlier; (b) Cooks distance is less than 1 for this data point, so the data point can be retained in the dataset.
